# Evaluation of Age Patterns of COVID-19 Mortality by Race and Ethnicity From March 2020 to October 2021 in the US

**DOI:** 10.1001/jamanetworkopen.2022.12686

**Published:** 2022-05-17

**Authors:** Irma T. Elo, Anneliese Luck, Andrew C. Stokes, Katherine Hempstead, Wubin Xie, Samuel H. Preston

**Affiliations:** 1 Deparment of Sociology, University of Pennsylvania, Philadelphia, Pennsylvania; 2 Population Studies Center, University of Pennsylvania, Philadelphia; 3 Department of Global Health, Boston University School of Public Health, Boston, Massachusetts; 4 Robert Wood Johnson Foundation, Princeton, New Jersey

## Abstract

This cross-sectional study examines age-specific COVID-19 mortality rates in the US from March 2020 to October 2021 by sex and race and ethnicity.

## Introduction

The disproportionate burden of COVID-19 mortality among older adults during the early stages of the pandemic is well documented.^1,2,3^ However, little is known about associations between subsequent developments (eg, vaccine availability) and the age pattern of mortality. In this study, we examined changes in age-specific COVID-19 mortality rates by sex and by race and ethnicity.

## Methods

All analyses for this cross-sectional study were conducted using provisional monthly data for March 1, 2020, through October 31, 2021, from the National Center for Health Statistics^4^ and monthly population estimates for 2020 and 2021 from the US Census Bureau.^5^ We included deaths for which an *International Statistical Classification of Diseases and Related Health Problems, Tenth Revision*, code of U07.1 was included among the stated causes of death on the death certificate. Analyses were stratified by race and ethnicity because prior research has documented pronounced racial disparities in age-specific COVID-19 mortality rates.^3^ Hispanic ethnicity and non-Hispanic race were based on single race coding on the death certificate and in the US Census Bureau’s population estimates. We calculated annualized COVID-19 death rates by 5-year age groups from 25 to 29 years to 80 to 84 years and for those 85 years or older. Additional details appear in the eMethods in the Supplement. The study used deidentified publicly available data and was therefore deemed exempt from review and informed consent by the University of Pennsylvania Institutional Review Board. This study followed the STROBE reporting guideline.

## Results

The Figure shows rates of age-specific deaths due to COVID-19 for 3 periods (March to June 2020 [first period], November 2020 to February 2021 [second period], and July-October 2021 [third period]) spanning the overall study period. Death rates were plotted on a logarithmic scale; a straight line would replicate the Gompertz curve in which death rates rise by a constant percentage for each additional year of age.^1,2^ For the first 2 periods, a straight line represented the age pattern reasonably well for non-Hispanic Black (hereafter Black) and non-Hispanic White (hereafter White) individuals, whereas the slope for Hispanic individuals declined at older than 70 years.

**Figure.**
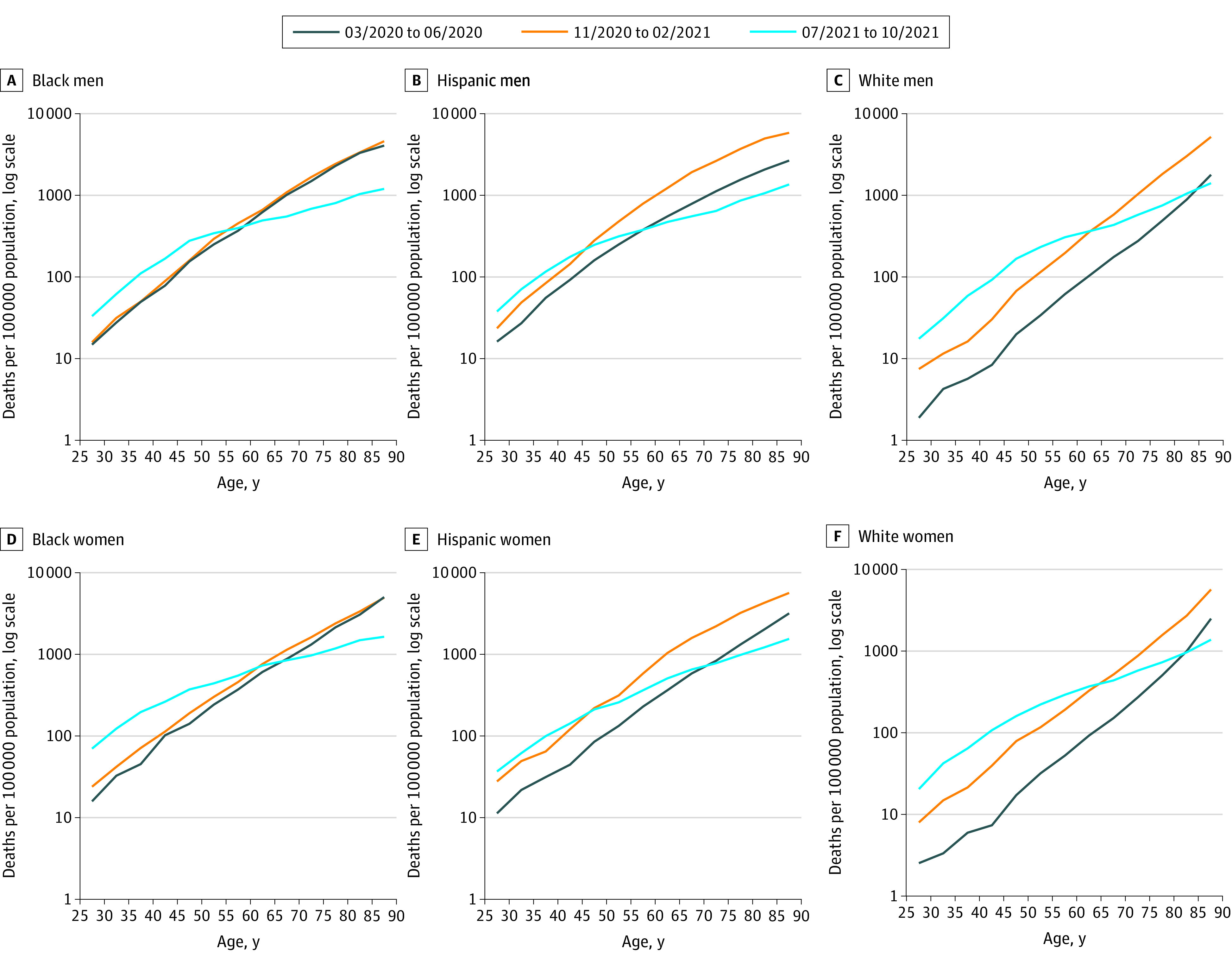
Age-Specific Death Rates Due to COVID-19 for Select Periods, March 2020 to October 2021

Across all sex and all racial and ethnic groups, there was a shift in the age pattern of mortality in the third period. Mortality fell by a higher percentage at older ages than at younger ages for all groups (ie, percentage declines ranged from 68% to 77% among those 85 years or older and 57% to 79% among those aged 80 to 84 years) (Table). Marked changes in age at death were observed among Black and White individuals, with the lines for the third period crossing over the earlier period lines (Figure). Mortality among Black and White men and women rose from ages 25 to 54 years from the second period to the third period (Table). For example, among Black men, the ratio of death rates at ages 25 to 29 years from the second to third period was 2.09; the respective ratios for Hispanic and White men were 1.61 and 2.35, respectively.

**Table.** Age-Specific COVID-19 Mortality Rates for Select Pandemic Periods, March 2020 to October 2021

Age group, y	Mortality rate per 100 000 population by race and ethnicity														
	Black					Hispanic					White				
	First period: 03/2020 to 06/2020	Second period: 11/2020 to 02/2021	Third period: 07/2021 to 10/2021	Ratio 1^a^	Ratio 2^b^	First period: 03/2020 to 06/2020	Second period: 11/2020 to 02/2021	Third period: 07/2021 to 10/2021	Ratio 1^a^	Ratio 2^b^	First period: 03/2020 to 06/2020	Second period: 11/2020 to 02/2021	Third period: 07/2021 to 10/2021	Ratio 1^a^	Ratio 2^b^
Men															
25-29	14.9	16.0	33.3	1.07	2.09	16.3	23.7	38.0	1.46	1.61	1.9	7.5	17.6	3.96	2.35
30-34	27.8	31.7	62.3	1.14	1.96	27.2	48.9	71.1	1.79	1.46	4.3	11.6	31.4	2.71	2.71
35-39	49.7	50.2	111.3	1.01	2.22	55.8	84.5	116.8	1.51	1.38	5.7	16.3	59.1	2.86	3.62
40-44	78.8	89.6	168.7	1.14	1.88	92.8	144.9	177.4	1.56	1.22	8.4	30.4	92.9	3.61	3.05
45-49	155.9	160.3	278.8	1.03	1.74	161.1	281.7	248.7	1.75	0.88	20.0	67.8	168.3	3.39	2.48
50-54	251.8	294.0	344.9	1.17	1.17	251.2	480.5	316.0	1.91	0.66	34.2	115.6	234.1	3.38	2.02
55-59	370.3	456.0	399.4	1.23	0.88	381.0	794.8	380.1	2.09	0.48	62.0	197.5	308.2	3.19	1.56
60-64	624.7	667.6	494.6	1.07	0.74	554.5	1234.8	474.1	2.23	0.38	104.2	358.5	364.9	3.44	1.02
65-69	1018.2	1095.9	553.2	1.08	0.5	791.6	1929.2	555.9	2.44	0.29	176.5	583.2	435.7	3.30	0.75
70-74	1494.6	1676.7	686.9	1.12	0.41	1127.6	2650.4	647.0	2.35	0.24	277.1	1040.7	580.5	3.76	0.56
75-79	2301.0	2436.3	807.5	1.06	0.33	1558.6	3718.1	866.7	2.39	0.23	492.6	1829.3	755.3	3.71	0.41
80-84	3323.2	3371.5	1042.1	1.01	0.31	2082.4	4993.3	1068.2	2.40	0.21	890.6	3030.7	1054.7	3.40	0.35
≥85	4084.7	4620.4	1205.1	1.13	0.26	2677.5	5862.1	1365.5	2.19	0.23	1792.5	5220.3	1415.4	2.91	0.27
Women															
25-29	8.0	12.3	37.4	1.53	3.05	5.6	14.3	19.2	2.55	1.34	1.3	4.3	11.5	3.29	2.65
30-34	16.9	22.0	67.1	1.30	3.06	11.2	26.0	32.8	2.33	1.26	1.7	8.2	24.3	4.71	2.95
35-39	23.8	38.1	109.0	1.60	2.86	16.3	34.4	53.9	2.11	1.57	3.2	12.0	37.6	3.76	3.13
40-44	55.2	61.5	147.4	1.11	2.4	23.4	65.9	78.0	2.82	1.18	4.0	22.8	64.1	5.73	2.82
45-49	77.3	105.2	211.1	1.36	2.01	45.8	122.8	117.8	2.68	0.96	9.6	46.6	96.9	4.86	2.08
50-54	135.2	169.6	252.1	1.25	1.49	72.6	177.1	144.8	2.44	0.82	18.2	69.9	136.2	3.84	1.95
55-59	210.9	262.2	316.9	1.24	1.21	127.8	336.4	206.0	2.63	0.61	30.5	116.7	180.8	3.82	1.55
60-64	352.0	443.7	425.2	1.26	0.96	206.1	613.2	291.5	2.98	0.48	55.1	204.9	231.2	3.72	1.13
65-69	515.0	673.6	495.0	1.31	0.73	337.2	951.4	378.3	2.82	0.40	91.5	327.9	275.2	3.58	0.84
70-74	783.1	970.0	570.2	1.24	0.59	488.3	1341.1	454.7	2.75	0.34	168.3	566.2	367.1	3.36	0.65
75-79	1297.8	1455.9	700.3	1.12	0.48	784.9	1980.2	579.0	2.52	0.29	319.7	1037.8	467.7	3.25	0.45
80-84	1883.2	2067.0	891.7	1.10	0.43	1231.5	2675.9	725.7	2.17	0.27	646.0	1824.2	624.8	2.82	0.34
≥85	3140.9	3091.7	985.2	0.98	0.32	1955.2	3553.8	926.6	1.82	0.26	1673.3	3924.2	904.1	2.35	0.23

^a^ Compares COVID-19 mortality rates from November 2020 to February 2021 with those from March to June 2020.

^b^ Compares COVID-19 mortality rates from July to October 2021 with those from November 2020 to February 2021.

Except at 85 years or older in the third period, Black and Hispanic individuals had consistently higher COVID-19 mortality rates than White individuals (Table). For example, in the third period, the mortality rate for Black women aged 50 to 54 years was 252.1 per 100 000 population compared with 144.8 for Hispanic women and 136.2 for White women. Mortality among men exceeded that among women for every age and every racial and ethnic group at all points.

Mortality change from the second period to the third period was similar by sex among Hispanic and White populations, but the mortality change experienced by Black women was worse than that experienced by Black men at every age (Table). Black women also experienced worse rates compared with Hispanic women.

## Discussion

Reductions in COVID-19 mortality among older populations are remarkable. From the second period through the third period, declines in death rates for the various racial and ethnic and sex combinations were especially large among those aged 80 to 84 years and those 85 years or older. Despite the availability of effective vaccines, COVID-19 mortality rose among younger adults. Possible factors underpinning these changing patterns are higher vaccination prevalence and less exposure to infection among older individuals.^1,6^ This advantage may have increased over time as younger individuals returned to work and other activities and the Delta variant emerged. Limitations included the use of provisional mortality data and the exclusion of other racial and ethnic groups owing to data quality issues. The rise in mortality rates among young adults underscores the value of increasing the lagging vaccination rate in this population.

## Notes

Supplement. **eMethods.** Statistical Analysis
