# Errors in Results, Table, and Figure

**DOI:** 10.1001/jamanetworkopen.2022.28383

**Published:** 2022-08-04

In the Research Letter titled “Evaluation of Age Patterns of COVID-19 Mortality by Race and Ethnicity From March 2020 to October 2021 in the US,”^1^ published May 17, 2022, a coding error affecting the calculation of the denominators of the death rates resulted in an inflation of the age-specific death rate estimates presented throughout the article. This error resulted in incorrect values presented in the Results as well as the age-specific death rates presented in the Table and the Figure. This article has been corrected.^1^
