# Supplementary material for: Evaluation of Age Patterns of COVID-19 Mortality by Race and Ethnicity From March 2020 to October 2021 in the US
Source: JAMA Netw Open. 2022 May 17;5(5):e2212686. doi: 10.1001/jamanetworkopen.2022.12686 (PMC9115616; doi:10.1001/jamanetworkopen.2022.12686)
Supplement: Supplement. — eMethods. Statistical Analysis [file jamanetwopen-e2212686-s001.pdf]

Supplementary Online Content

Elo IT, Luck A, Stokes AC, Hempstead K, Xie W, Preston SH. Evaluation of age patterns of COVID-19 mortality by race and ethnicity from March 2020 to October 2021 in the US. *JAMA Netw Open*. 2022;5(5):e2212686. doi:10.1001/jamanetworkopen.2022.12686

eMethods. Statistical Analysis

This supplementary material has been provided by the authors to give readers additional information about their work.

© 2022 Elo IT et al. *JAMA Network Open*.

eMethods. Statistical Analysis

All analyses were conducted using a combination of publicly available data from the National Center for Health Statistics (NCHS) and the United States Census Bureau. Provisional monthly death counts were obtained for March 2020 through October 2021 for the US population aged 25+ by 5-year age groups (25-29, 30-34, ... 80-84, 85+), sex, ethnicity and single-race coded categories for race.[4] The analyses included all deaths assigned to Covid-19 either as an underlying or a contributing cause of death on the death certificate using the ICD-10 code U07.1. Monthly population estimates for 2020 and 2021 by age, sex, ethnicity, and single-race coded race were obtained from the Census Bureau's monthly national-level population estimates.[5] We limited the analyses to Hispanic, non-Hispanic Black and non-Hispanic White populations because of potential data quality issues in matching death records and census data for other racial groups. Annualized age-specific death rates by race and ethnicity and sex were calculated by dividing the total number of deaths in each period by the total person-years lived in that period for selected four-month periods by five-year age groups roughly corresponding to March-June 2020, November 2020-Februrary 2021, and July-October 2021, reflecting the high mortality rates in March-June 2020, the winter mortality peak in November 2020-February 2021, and the arrival of the Delta variant in July 2021-October 2021.

© 2022 Elo IT et al. *JAMA Network Open*.
